# Yoga as an adjunct treatment for eating disorders: a qualitative enquiry of client perspectives

**DOI:** 10.1186/s12906-024-04514-1

**Published:** 2024-06-24

**Authors:** Jennifer O’Brien, Shane McIver, Subhadra Evans, Eleanor Trethewey, Melissa O’Shea

**Affiliations:** 1 School of Psychology, Faculty of Health, Deakin University, Melbourne, Australia; 2 School of Health and Social Development, Deakin University, Melbourne, Australia

**Keywords:** Yoga, Eating disorders, Psychotherapy, Mental health

## Abstract

**Background:**

This qualitative enquiry explores the experiences and perspectives of individuals with an eating disorder (ED) regarding their perceptions of yoga as an adjunct intervention to psychotherapy. It also explores the feasibility, acceptability, and safety of yoga from their perspectives.

**Methods:**

This study used a practice-based evidence framework and employed semi-structured interviews with 16 females with an ED. Participants were asked about their perspectives on the use of yoga as an adjunct intervention in ED recovery, perceived risks and what factors supported or hindered engagement. Thematic template analysis was used.

**Results:**

Three topic areas were elaborated. The first included participants’ perceptions of how yoga enhanced their ED recovery. The second included how and when participants came to find yoga in their ED recovery. The final topic explored factors that supported participants with ED to engage in yoga. These resulted in the development of guiding principles to consider when designing a yoga intervention for EDs.

**Conclusions:**

This study adds further to the emerging evidence that yoga can bring complementary benefits to ED recovery and provides a biopsychosocial-spiritual framework for understanding these. Findings provide an understanding of how yoga programs can be adapted to improve safety and engagement for people with an ED. Yoga programs for people with EDs should be co-designed to ensure that the physical, social, and cultural environment is accessible and acceptable.

**Supplementary Information:**

The online version contains supplementary material available at 10.1186/s12906-024-04514-1.

## Plain english summary

Recent research has explored the benefits of yoga for individuals with eating disorders (EDs). However, only a few studies have interviewed individuals about their perspectives on using yoga within their ED recovery. This study interviewed 16 individuals with an ED to obtain their perspectives about using yoga as a therapeutic treatment in their recovery. Participants perceived multiple benefits of how yoga enhanced their recovery. They reported their views on how to incorporate yoga in ED treatment approaches to decrease potential risks. This study offers unique insights into how yoga may be safely applied to the care of people with an ED to enhance their treatment and recovery.

## Background

Eating Disorders (EDs) are a set of psychiatric diagnoses characterised by an unhealthy preoccupation with eating, exercise, and body weight or shape [1]. They are associated with a broad range of negative outcomes, including medical complications, major disruptions in cognitive, emotional, and social functioning [2], and significant impacts on quality of life [3, 4]. Presently, EDs represent a major public health concern, with an estimated 4% of the Australian population affected [5], with research showing a rise in presentations since Covid-19 [6, 7]. Current treatment for EDs includes a multi-axial approach, including medical, psychiatric, and psychological interventions [5] with transdiagnostic approaches such as family-based therapy (FBT) (primarily for younger people) [8] and enhanced cognitive behavioural therapy (CBT-E) (for adults) demonstrating positive results [5, 9–11]. Nonetheless, treatment research outcomes indicate high rates of dropout, modest rates of recovery, and high rates of relapse [5]. As such, some ED services have incorporated complementary interventions, such as yoga [12], that aim to facilitate a mind–body connection in the hope that this may support recovery [13].

Yoga typically involves a combination of breathing practices, gentle physical poses, and meditation [14]. Research examining yoga suggests it has a promising effect on ED symptomatology [12, 13, 15]; such as improving psychological factors including body responsiveness and awareness [16], interoception and embodiment [17–19], mindfulness, self-compassion, self-efficacy [20] body satisfaction, body appreciation, and body image [21–24]. Despite this, given the lack of research, currently, yoga is not recommended as a stand-alone treatment but as an adjunct intervention for the treatment of EDs [12, 15].

Research indicates that people with lived experience of EDs perceive yoga as helpful for recovery [25]. However, only a small number of qualitative studies published to date examine these perceived benefits. One qualitative study employed in-depth interviews (*n* = *16)* to understand the experiences of using yoga to support ED recovery for women with Anorexia Nervosa (AN); finding that yoga enhanced embodiment, leading to increased feelings of empowerment, enhancing ED recovery [26]. In their subsequent publication, Pizanello [27] analysed their results further, explaining that the enhanced embodiment improved participants’ interoceptive awareness and improved mindfulness and emotion regulation. In another study, [28] interviewed a single individual with AN finding yoga enhanced a new sense of body acceptance, awareness, and safety, allowing them to process traumatic memories safely. Both studies focussed on AN only, and questions relating to yoga's perceived risk or safety were not explored. Two further studies used qualitative methods as part of a mixed methods evaluation of novel yoga programs for people with EDs [29–31]. Diers et al., [29] piloted a yoga program for 67 people diagnosed with an ED using mixed methods with quantitative data showing decreased body image concerns. The qualitative data suggested that the yoga program improved participants’ self-acceptance, self-awareness, confidence, and emotional and physical strength, and that group discussion enhanced the experience of embodiment through verbal processing and peer-based support. However, along with these perceived benefits, participants reported that at times they had negative experiences with yoga due to self-judgement, vulnerability, and confrontation of uncomfortable feelings McIver et al., [30, 31*] piloted a yoga program for 25 people with Binge Eating Disorder (BED) which resulted in a decrease in BED symptomatology with qualitative data indicating that participants felt more positively connected to their bodies, and food, resulting in feelings of self-empowerment [30, 31]. Qualitative data in this study was limited to self-report diary entries, and participants were not asked about the feasibility or safety of the yoga program. Adding to this picture are two qualitative studies examining yoga's impact on body image in the general population [24, 32]. These studies found yoga led participants to feel empowered, which improved body image and acceptance but were coupled with findings that at times yoga had a negative impact on individuals as it could encourage comparative, negative and perfectionist thinking [24, 32].

Given that current literature indicates that people with an ED report both positive and negative experiences when engaging with yoga, an examination of yoga's acceptability, feasibility, and safety is a critical next step. This is particularly important as yoga is commonly used in contemporary ED services [12] As such, this study employs a Practice-Based Evidence (PBE) framework [33, 34] where in the perspectives of people with an ED as to the acceptability, feasibility and safe of yoga as an adjunct intervention to ED treatment is sought using semi-structured interviews. The study is the second of a two-part qualitative enquiry, with the other focusing on mental health clinicians' perceptions [35].

The research questions for this study are as follows:

### From the perspectives of people with an ED,

1. What are the perceived benefits of yoga as an adjunct intervention for ED treatment?
2. What are the perceived risks or safety issues of yoga as an adjunct intervention for ED treatment?
3. What are the factors that support or hinder engagement with yoga as an adjunct intervention for ED treatment?

## Methodology

### Study design

A constructivist realist approach was taken, which argues that research is not independent of the clinical researcher’s perspective but that meaning is observed, described, and even co-created [36]. Specifically, a PBE framework was adopted to integrate clinical expertise with systematic research evidence [33]. Qualitative enquiry was used to draw out stories and understand experiences [37], with semi-structured interviews being used to capture these rich stories [38].

### Recruitment

The study was advertised on Eating Disorders Victoria’s (a not-for-profit peak body) website and social media pages and at a regional ED service in Victoria, Australia. This advertisement explained that the study was recruiting a broad range of participants with a lived ED experience to understand their perspectives on yoga, noting that experience with yoga was not a requirement of participation. Interested participants completed an online consent form and were provided with a plain language statement. Once consent was obtained participants were contacted via email by the researchers and offered an interview via Zoom or telephone. The study was approved by Barwon Health Research Ethics, Governance and Integrity Unit [RHEA-75618] in September 2021.

Purposive sampling procedures were used and participants were deliberately invited due to their unique insights related to their experience with an ED [39]. Inclusion criteria included that the participant self-identified as having a diagnosed ED and had received treatment for this. The only exclusion criteria was prospective participants aged under 12 years. Recruitment was not limited to individuals who had experienced yoga but was open to anyone with an ED diagnosis and interested in participating.

### Participants

Interviews were completed with 16 female participants who had been diagnosed with an ED and had received psychological treatment for it. Participant diagnoses ranged from AN (*n* = 9), Atypical AN (*n* = 1), AN (restrictive subtype orthorexia behaviours) (*n* = 1), BED (*n* = 2) & BN & AN (*n* = 2) and one participant reported that they were not given a formal diagnosis (*n* = 1). We asked participants to describe their ED journey, which indicated there was a broad range of experience. Some reported they were recently diagnosed and in active treatment, others reported that they were recovered, and some reported that despite symptom resolution, the ED would always be with them. Participants were or had been engaged in various ED services (public, private, community and inpatient) within Australia. All but one participant volunteered for the study as they had used yoga alongside their ED treatment and felt that it had enhanced their recovery. However, all had experience and understanding of what yoga was.

### Procedure

Interviews occurred over an extended timeframe due to initial recruitment difficulties (September 2021 to August 2022). Semi-structured interview schedules were used to provide consistency across all interviews while allowing participants to elaborate and tell their unique stories [38]. All interviews were completed on Zoom, given a unique identifier code, audio recorded, and transcribed. Any reference to names or places was omitted to ensure confidentiality. Participants were free to withdraw from the study at any time without consequences and were given a $30 gift voucher to reimburse them for their time.

### Data collection and analysis

The interview guide had two streams of questions to capture a diversity of experiences with yoga [40]. One stream catered to individuals who had had some experience with yoga and the other to those who were new to yoga. For those that had used yoga alongside ED treatment approaches, questions such as: 1) What brought you to yoga in the first place? 2) Did you find that yoga brought any benefits to your health? 2) Are there any complementary benefits that yoga brings to your current psychological treatment? 3) Do you have any concerns about engaging in yoga? For those with little experience with yoga, questions such as: 1) Do you think yoga could bring physical and mental health benefits? 2) Would you have any concerns about engaging in yoga?

Data were analysed using template analysis as it provided a guiding set of procedures [41] that can be used in a top-down qualitative analysis. Template analysis is a practical approach that emphasises the use of hierarchical coding. It balances a relatively high degree of structure in the process of analysing textual data with the flexibility to adapt it to a particular study [41, 42]. Template analysis has been successfully used with PBE research, particularly when the study aims to understand an intervention’s acceptability, feasibility and safety in clinical populations [43–45]. Central to the approach is developing a coding template, which enables a priori themes to be selected to structure the analysis [42].

After completing the interviews, the researchers reviewed the data to familiarise themselves with it. An initial subset of data (*n* = 5) was taken by researchers (JO’B & SMcI) who commenced preliminary coding. This process included coding data in relation to the research questions and priori themes and grouping data where it was the richest [42]. Mind mapping was used to link codes of similar concepts that had a relationship with each other, followed by an iterative process that identified distinct themes and relevant sub-themes. Quotes were noted where the data was the richest, highlighting the themes identified. At all stages of coding and theme development, to ensure research rigour, documentation was kept, ensuring that the evolution of themes was clear and traceable [36]. The resulting thematic structure defined the initial template codes, which was further developed through the coding of another sub-set of interviews (*n* = 5). All interviews were then reviewed by another researcher (MO’S) to ensure the template codes reflected the data. Researchers reviewed each other's codes and discussed the similarities and differences throughout the process before identifying and agreeing on themes and sub-themes [42]. This resulted in the final template codes and themes that were grouped into topic areas, which was then used with the remaining datasets, confirming that these were a comprehensive and rich representation of the data.

## Results

We identified three topic areas: (a) yoga’s capacity for healing and transformation in ED recovery; (b) when and how participants came to find yoga within their recovery and (c) what factors supported them to engage in yoga.

## Yoga’s capacity for healing and transformation

**Fig. 1:**
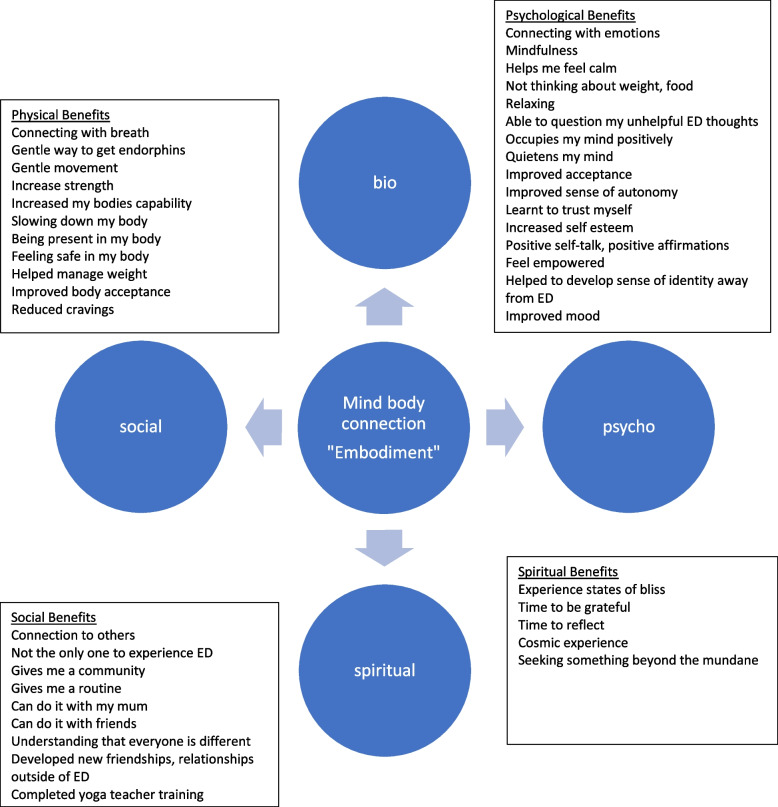
How yoga helps me

Participants saw multiple benefits of yoga for their ED recovery, spanning physical, psychological, social, and spiritual domains (See Fig. 1). These were frequently described as overlapping, whereby one benefit was viewed as leading to another. For some participants, yoga was identified as the key to their recovery, describing transformative experiences with the practice*.*

*"It’s helped the attitude I have towards my body, it’s made me more aware of a different way of seeing my body, instead of seeing it from the outside, how it looks from the experience of the inside, and so whenever I feel self-conscious about my body in public, I try to notice how it feels instead." (Participant 10 (AN)).*

Some participants reflected that they previously experienced gaps in their current ED treatment, noting that it was heavily focused on nutrition and talking, neglecting how to relate positively to the body. Comparatively, they described yoga as being different in important ways, emphasising body connection and acceptance. “*I really loathed my body, but the yoga taught me to love my body and how strong it is and what it's able to do.” (Participant 6 (AN)).*

Several participants remarked that yoga was an approach that worked for their bodies and minds. In acknowledging “*the body and the mind is ONE thing, they’re not separate things” (Participant 11 (AN))*; they recognised that this unity was vital to recovery *“in your psychology treatment for your ED, you have to have something that teaches you how to be okay with how your body feels, 'cause most of the time you spend time trying to get out of it.” (Participant 11 (AN)).* Many participants described how yoga provided something different and complementary to psychotherapy. The yoga was described as providing an experiential and somatic method to explore their ED symptoms safely, which they felt psychotherapy could not do alone. For some, yoga was seen to be more beneficial for their recovery than psychotherapies such as CBT.

*"Like if I was able to do those CBT skills, as I'm sitting here doing like absolutely nothing, I wouldn't be having these issues, but for me, the yoga was a way for me to physically force myself into it." (Participant 8 (AN subtype orthorexia behaviours)).*

### Physical benefits

Participants reported that yoga improved their body’s physical capabilities and helped them to gain physical strength; *"a pose that was really hard a month ago now seems a little bit easier, you can hold things a little bit longer and you kind of notice your strength improving which is satisfying” (Participant 13 (BED)).* Many participants talked about experiencing a difficult relationship with their bodies and that re-engaging with movement was a safe way to reconnect with their bodies, leading to a unique opportunity to explore the discomfort associated with their bodies. One participant reported:

*"You'll be in a stretch and you'll feel that discomfort, but then on top of that, you might feel like your stomach or your ribs on your leg and stuff like that, getting used to that uncomfortableness but it was temporary. I could choose to come out of that stretch and that was really good for me." (Participant 8 (AN subtype orthorexia behaviours)).*

Several participants also reported that yoga helped them to manage their relationship with eating, weight and cravings *“I went to yoga to relax and occupy my mind so that I am not thinking about what I’m going to eat. The mindfulness aspect helped because a lot of my eating is because of boredom or pain.” (Participant 5 (BED)).*

### Psychological benefits

Participants talked about how yoga allowed them to develop mindfulness and relaxation skills. When participants engaged with their bodies via yoga, this connection allowed them to experience a psychological break from distressing ED cognitions that were normally present.*”it just takes me out of that and it gives me a different mindset, which is nice not to hear the ED all the time” (Participant 9 (AN)).* Viewing distressing ED cognitions from a distance gave participants space to appraise and challenge these. Participants talked about how this led to feeling empowered, improved their self-esteem, increased positive self-talk, improved body acceptance, and helped them to learn to accept and trust themselves. One participant described how this then allowed them to explore a new sense of self separate from the ED.

*"It just kinda makes me calm and it makes me... I'm not so focused on how I look, what I eat, that sort of thing, and it just... It reminds me of what I actually want out of life. Like I wanna be happy and calm and mindful and not worried about weight and what I’m eating." (Participant 4 (BN & AN)).*

### Social Benefits

Participants talked about yoga’s social benefits, such as a sense of community, and that they were not experiencing the ED alone. Participants talked about how these social connections promoted the feeling of being accepted and belonging. *“I feel a connection to other people with EDs without having to talk about it" (Participant 2 (Atypical AN)).*

Participants gained a sense of satisfaction from these social connections as described.

*"I think the diversity of people you find in there helps, the yoga studio has some old person, some guy that's recovering from a knee injury...and there is just anyone and everyone, it kinda feels like a nice space where you don't have to be self-conscious." (Participant 11 (AN)).*

Some participants described yoga as a meaningful hobby connecting them with people and a community. Two participants reported that they went on to become qualified yoga teachers.

### Spiritual benefits

A few participants reported that yoga provided them with spiritual benefits, which was important in their recovery. For example, yoga gave one participant a feeling of gratitude and another an opportunity to seek something beyond the mundane. One participant explained that previously the only way to experience positive emotional states was through starvation whereas yoga now provided another avenue.

*"You're reaching a higher state and not only is it not destroying you, it’s building you up, you're tapping into different frequencies you did before, it's centrally like a cosmic experience……… Essentially, we are spiritual beings, …..it kind of makes sense that you're trying to seek something beyond mundane living." (Participant 7 (AN)).*

The participants that described spiritual benefits saw these as very important as yoga helped them connect deeper with a sense of themselves. *“I found it to be really relaxing and help with mindfulness with the body, like your positive spirit, you're going into another trance with yoga.” (Participant 5 (BED)).* This further provided distance from ED pathology, allowing this participant to connect with their sense of identity which they saw as integral to their recovery.

## My yoga journey: how I found yoga, the timing counts

Participants found yoga at different times in their ED recovery, impacting their experience and attitude towards engaging with it. Several participants engaged in yoga whilst as an inpatient. These participants talked about how yoga was a safe way to start moving again after being physically compromised. These participants tended to start with gentle yoga. They built up their practice over time. Yoga was seen as a complementary intervention to psychotherapy and benefits were observed early in their recovery.

*“We had to do it as one of our groups, and I just learnt that as it’s a good way of trying to practice movement without unhealthy movement, it’s movement where you are taking care of your body.” (Participant 12 (BN & AN)).*

Other participants talked about how treating health professionals discouraged movement practices until medical stability was established. These participants tended to find their own yoga class in the community with less guidance or support which made it more challenging for participants to select an appropriate class that worked for them. Some participants in this group acknowledged that initially, they tried faster more intense yoga, hoping that the yoga would result in calorie expenditure.

*"One of my health professionals was really supportive of it, but then she found out exactly what it was, and she was sort of a bit like, Okay, maybe we need to back up on this and figure out a more appropriate way to do it." (Participant 8 (AN restrictive subtype orthorexia behaviours)).*

These participants reported that over time, as they experienced the benefits of yoga, they could reduce the intensity and move to more mindful practices.

A few participants with chronic ED symptoms that found yoga much later in their recovery, looking to yoga as an alternative treatment option. In contrast to those participants who found yoga earlier in recovery, these participants felt that earlier ED treatment interventions had failed to address key issues related to their illness. They saw yoga as an intervention that addressed the ED differently. It was a method to experience and understand their body ‘from inside, out’ allowing them to develop somatic ways to address their ED. *“I feel that in recovery, we need to be looking deeper… And that's what yoga does. It really starts to sort of nurture, basically that inner child that's been completely wounded.” (Participant 14 (AN)).*

Overall, offering yoga earlier and providing multiple opportunities to engage, was seen to improve the likelihood of finding yoga to be of benefit in recovery. Participants talked about how important it was to have multiple opportunities to engage with yoga as not all took it up when it was first introduced.

## Factors that support engagement with yoga

Participants identified factors that supported a safe engagement with yoga and factors that felt unsafe or aversive (See Table 1). Most expressed a view that if yoga is not designed for people with EDs, it could be harmful and exacerbate ED symptoms, such as negative and critical thinking, body dissatisfaction, and excessive exercise. Participants talked about the importance of the physical and social environment, the type of yoga and who facilitates it as critical factors in enhancing engagement.

**Table 1:** Factors that impact engagement with yoga for individuals with an eating disorder

Physical Environment	Social Environment	Yoga Protocol	Teacher
Quiet space	Small group	Multiple options for different poses	Warm and inviting
Low lighting	Similar demographics	Multiple options for different pace	Mental health clinician present as well would help participants feel safe
No mirrors	(ED, age, gender, should be considered)	Slow yoga generally seen as helpful	Understanding Of E’s important
Options for in person and online	Loose clothing encouraged	Hatha yoga generally seen as helpful	Trauma informed approach
Comfortable mats and props	ED specific class	Yoga class themes linked to ED topics	Invitational language
Ability to do in group and at home practice	Having family or carer present for support	Building up the yoga practice over the sessions	Minimise triggering words/phrases associated with ED (such as how bodies should move/feel)
Aromatherapy helpful	Participants at similar stage in recovery helps	Emphasis is on fun, play and curiosity	Go through poses before practicing
Close to home and low cost	Participation is voluntary (not mandatory)	Body positive yoga protocol	Ensuring teacher understands individual’s physical capacity
If is at an ED service, good to have separate room to where other treatments occur	Knowing that participants may rest during practice	Setting intentions at beginning of yoga	One on one with teacher before starting the group
Yoga mats positioned in a circle (to reduce comparative thinking)	Body positive messaging helps build social environment	Slowly building up shavasana so that it’s not overwhelming	Ensuring teacher does not touch students or engage in corrections
Enough space between participants	Capacity to leave or engage as needed by participants	Protocol should be tested with larger bodies to ensure poses are accessible	Body positive messaging important

### Physical environment

Consistent among participants was the view that a warm and inviting physical environment was critical. Multiple participants noted that a yoga environment was ideally quiet, with low lighting and no mirrors. Access to classes was important; those attending in the community talked about the difficulty in finding classes that were appropriate and close to home. Some participants noted that online options meant they could access yoga at home and that this mode also helped them manage self-judgement arising from comparing themselves to others. *“I prefer to do it alone at home, just because of my own anxiety, and feeling out of place and worried about people like judgement and stuff. Which is a lot of my own anxiety” (Participant 4 (BN&AN)).*

### Social environment

The social environment was important, with participants reporting feeling self-conscious about their clothing and physical abilities and wanting to fit in. This negative self-evaluation was less likely to occur in small-group yoga with people of similar age and gender. Participants emphasised the need to wear loose and comfortable clothing, with many holding negative feelings about the yoga tight clothing fashion expectation. When the social environment was experienced as negative, this increased an individual’s comparative thinking and self-judgement. *“I've seen other people in the waiting room before and it's made me think if you're really bad, I shouldn't be there 'cause they are actually underweight.” (Participant 2 (Atypcial AN)).*

### Type of yoga

The type of yoga was very important to participants, with many suggesting the need for the facilitation to be slow and gentle, even more so early on. As discussed earlier, there was a discrepancy among participants in that some participants drawn to faster movement found it difficult to engage in slow yoga early in recovery. One participant that did not use yoga as part of their ED recovery (all other participants did), described how the gentleness of the yoga put them off *“It's just too peaceful. [laughter] I'm not a very peaceful person. It's very quiet, I'm not a very quiet person.” (Participant 1 (AN)).* Other participants noted that it took time to understand the somewhat paradoxical benefits of yoga – in that a gentle movement practice previously viewed as a relaxation activity, could promote strength: *"I feel like quite energized afterwards, and it's like the whole nature of it is being gentle on your body and listening to it while also feeling strong, noticing, building strength each time you do it and getting better at it. It's nice.” (Participant 10 (AN)).*

Some participants described the importance of offering options for poses, ensuring that poses could be modified for those in larger or differently abled bodies and inviting participants to move in a way that felt comfortable for them. One participant described how her body was not able to engage with certain poses, but the teacher did not offer any suggested modifications leaving her to feel disheartened. *“You get the sense that they almost do not know what to do with a bigger body.” (Participant 13 (BED)).* Similarly, one participant described how important bolsters, blocks and other props were for her to be able to engage without pain. Most participants believed that specific ED yoga classes would help them feel more comfortable attending, noting that knowing other participants had similar experiences would help. Some suggested that using themes each week could allow participants to process and think about different aspects of ED recovery.

### Role of the yoga teacher

Participants emphasised the role of the yoga teacher in supporting or detracting from their experience and was instrumental in building a warm and inviting group experience. Many suggested that having both a yoga teacher and mental health professional present would increase comfort and safety. Education about the complementary benefits of yoga for ED recovery and encouragement from a health professional was seen as key to supporting safe engagement. Many thought it important that the teacher understood EDs well as thoughtful instruction could reduce the striving and comparative thinking that was present for many. The language used was important to participants, who suggested teachers minimise the use of ‘triggering’ words (such as when referring to body parts or ways bodies were ‘meant’ to move or feel) as this can reinforce the idea that there is a right or wrong body or a way to do yoga.

*"The voice in my head was incredibly loud, being incredibly critical, and it was very difficult, but with the right teacher and the right yoga script, it's very healing in the sense that what I experienced was being able to surrender to the now and practice acceptance." (Participant 16 (Diagnosis unknown)).*

Participants also talked about the importance of the teacher acknowledging that everyone’s body is different to increase body positivity and acceptance.

## Discussion

This qualitative enquiry explored the experiences and perspectives of individuals with an eating disorder (ED) regarding their perceptions of yoga as an adjunct intervention to psychotherapy. It also explored the feasibility, acceptability, and safety of yoga from their perspectives. Results explored three topic area’s; yoga’s capacity for healing and transformation, when and how yoga was found, and what factors supported engagement with yoga.

Participants identified a range of physical, psychological, social, and spiritual benefits of yoga in their recovery. Accordingly, the reported benefits are consistent with previous yoga research, which found that yoga can provide biopsychosocial-spiritual benefits. Physical and psychological benefits such as managing weight gain and cravings [46], improving physical strength and capacity [47], body satisfaction, body appreciation and body image [21, 22, 24, 29, 48], mindfulness, self-compassion [20], self-efficacy [49], self-regulation [50], empowerment [30, 31], motivation [49], connection [51], psychological flexibility [50], positive affect [52] where all reported by participants. Furthermore, participants reported that yoga provided an opportunity for developing social connections and a sense of belonging [13] and an opportunity for deeper spiritual reflection [32].

Overall, participants reported that yoga was a helpful adjunct to their psychological therapy, providing somatic and experiential ways to enhance recovery. The limited available literature suggests that there are benefits in using yoga as an adjunct therapy for a range of mental health diagnoses [53] and may enhance engagement in psychotherapy processes [52, 54]. Yoga’s multiple techniques can provide an experiential platform [55] promoting introspection, cognitive, emotional, and behavioural changes [56], making it functional and transdiagnostic in its approach [57].

Importantly, participants endorsed the mind–body approach of yoga as key to recovery, explaining how benefits were interrelated and linked. Participants identified that the physical movement of yoga represented an essential process for safely reconnecting with their bodies and enhancing embodiment. Participants described how safely connecting with their bodies improved mindfulness and relaxation, allowing distance from their ED thoughts and an opportunity to appraise and challenge these.

Previous qualitative research has found that yoga has enhanced embodiment [58] and supported ED recovery in various ways [59]. For example, Pizanello [26] found that embodiment led to a new sense of empowerment (whereas power and control had only been previously experienced through maladaptive starvation behaviours), which allowed a deeper connection to the authentic self, supporting self-identity development necessary for ED recovery. Pizanello [27] then re-analysed this data through the lens of psychodynamic theory (D.W Winnicott's Object Relations Theory), attributing embodiment with improved interoceptive awareness and emotion regulation skills, as the mechanism that enhanced ED recovery. Osterman et al., [28] attributed embodiment with improved body acceptance, awareness and safety allowing one to process traumatic memories and ultimately recover.

In this study, participant responses reinforced previous findings that embodiment was the catalyst that facilitated further recovery processes (psychological, social, and spiritual) necessary for ED recovery. This study adds to existing evidence as additional pathways were identified for how yoga’s capacity to enhance embodiment can improve well-being [19, 58, 59] for various ED presentations. For example, participants with AN described how yoga helped them to slow down, improve mindfulness and distance themselves from critical thoughts. In contrast, participants with BED described how yoga supported healthier habit development, such as developing regular exercise patterns and healthier eating choices. In these examples, mindfulness practice was integral in enhancing motivation, which in turn can support the implementation of positive behaviour change interventions [60].

This study also adds to the existing literature on the social and spiritual benefits of yoga. These recovery domains are well-noted as important in ED recovery [61–63] but are not routinely addressed in psychotherapy. Participants described social benefits such as increased social connections, a sense of belonging, giving them new roles, and hobbies [64]. Participants described spiritual benefits such as an enhanced connection to identity, purpose, and overall sense of spirituality. These findings contribute further to our understanding of the prominent yoga model of health [65]. This also supports the rationale that in adjunct with psychotherapies such as CBT-E [57] yoga may be of additional value [66], as it addresses all biopsychosocial-spiritual elements [67, 68] of recovery in a way that psychotherapies cannot alone.

This research provided insights into the benefits and challenges of engaging with yoga throughout the stages of recovery. Findings suggest that those offered yoga earlier were more likely to engage. When yoga was provided by a health service, education about yoga’s benefits and risks was provided, and the yoga focussed on ED recovery, participants felt that they were more likely to use yoga helpfully. A barrier to including yoga in ED services includes a lack of research into safe guidelines for the use of yoga and clinical staff concerns that yoga may exacerbate ED symptoms [69]. These findings suggest that ED services engage clients in discussions about the potential benefits and risks of using yoga early in treatment, as adequate education enhances outcomes and decreases risks. Our study findings indicated that when guidance from health professionals was lacking, participants sought out yoga classes themselves, which often perpetuated maladaptive ED cognitions. For example, participants with AN described initially seeking out faster yoga to burn calories. Previous qualitative research has identified potential risks associated with yoga as it can trigger comparative thinking and body checking [24, 26]. When yoga teachers did not provide appropriate adaptations, participants with BED in a larger body described not being able to engage in certain poses as their bodies were unable to or experienced physical pain. The results of this study identified how these potential risks were seen to be mitigated or overcome when the yoga teacher has knowledge and training in EDs and provides thoughtful facilitation.

Study findings also indicated that participants who found yoga later in their recovery felt strongly that they missed opportunities for ED recovery, as these participants perceived psychotherapy alone as inadequate. Taken together with research indicating that early intervention can enhance ED recovery [70–73], and that yoga is an effective ED prevention intervention [48, 74] and can reduce anxiety when used in early intervention with a range of mental health disorders [75, 76], it is recommended that ED services offer it early in recovery [77]. There is a body of evidence that suggests that over time, EDs can become more entrenched through functional deterioration, neuroadaptation, and the development of chronic behaviour patterns [73, 78, 79] further reinforcing the importance of offering a range of transdiagnostic treatment options early in recovery.

This research identified a range of factors that participants viewed as supporting the establishment of a safe and inviting yoga environment. Many of these factors, such as using trauma-informed language, focusing sessions on themes related to ED recovery, and creating a safe and inviting environment, reflect elements included in yoga protocols for EDs that have been recently trialled [29, 80]. Participants reported that if the yoga was designed for ED recovery and offered in collaboration with the ED service, having both a yoga teacher and mental health professional present would provide further confidence that the yoga would be used to promote recovery. The yoga teacher was seen as essential in building a supportive environment, acknowledging different bodies in the room, offering multiple poses and options for different bodies and being aware that critical or comparative thinking can occur. Given the diverse range of bodies and subsequent need for a range of adaptations and accommodations to support yoga participation within contemporary ED services [81] participatory research methodologies are recommended to adapt and design complementary yoga programs for ED treatment to ensure end-user participation, engagement, and safety [82, 83]. This research points to the benefits of offering in-house adjunctive yoga for ED recovery, to provide education and an opportunity to experience how yoga can enhance embodiment, provide biopsychosocial-spiritual benefits and support ED recovery along with decreasing any potential risks. Taken together, these findings provide potential guidance for practitioners and services seeking to integrate yoga programs within current treatment programs. Drawing on PBE, qualitative research can be translated into intervention design objectives and subsequent guiding principles [84]. Table 2 describes guiding principles for designing an adjunct yoga intervention for ED recovery and may be a useful starting point for clinicians and researchers interested in future implementation of yoga-based ED recovery programs.

**Table 2:** Guiding principles for designing an adjunct yoga intervention for ED recovery

Intervention design objective	Key intervention features
*To offer a yoga practice to complement/enhance the goals of ED psychological treatments *	• Consider themes for sessions related to ED recovery • *Encourage participants to explore the impact of yoga on ED recovery with their treating ED clinician * • Provide education about possible benefits of yoga
*To promote safe movement / exercise practice *	• Ensure yoga is slow and mindful • *Ensure a mental health clinician is present during yoga sessions for psychological support as needed * • *Use trauma informed language and practices in intervention design * • Ensure yoga facilitator is educated about EDs
*To promote sense of social connectedness *	• *Consider offering yoga in a supportive group setting * • *Use principles from group theory to support the development of safety and cohesion between participants*
*To promote choice and autonomy in recovery planning *	• *Use co-design principles when developing group to consider needs of participants * • *Offer yoga at multiple times throughout recovery journey in multiple settings *
*To promote accessibility for different bodies *	• *Consider the physical environment (including props) and offer range of poses * • *Ensure yoga practice is tested and feedback gained to improve accessibility for participant’s needs *

### Limitations

A limitation of this study was the lack of demographic data collected from participants; subsequently, factors such as ethnicity, race, and age could not be considered. Furthermore, most participants volunteered for the study as they found yoga helpful to their recovery. Future enquiry may benefit from separately recruiting participants with positive and negative perspectives, including the perspectives of participants with little to no yoga experience. Additionally, more than half of the participants were diagnosed with AN. Future research would do well to have more participants for each ED diagnostic criteria to further contrast similarities, differences, and clinical implications.

## Conclusion

This PBE research offers a biopsychosocial-spiritual framework for understanding yoga’s benefits to ED recovery. Findings from this study highlight further understanding of how yoga can enhance embodiment and ED recovery processes for a range of diagnoses. Participants from this study found that yoga enhanced embodiment which was the catalyst to engage with psychological strategies, such as mindfulness, cognitive appraisal and challenging, supporting ED recovery. Other findings indicate that offering yoga early is helpful and more likely to be safe when supported by health professionals. Yoga programs should be co-designed to ensure the physical, social, and cultural environment is accessible and acceptable. This study adds further weight to the emerging evidence that yoga can bring complementary benefits to ED recovery.

### Supplementary Information

Supplementary Material 1.

## Data Availability

A supplementary file attached contains raw data of all qualitative interviews along with analysis and final template documents.
