# Supplementary material for: Yoga as an adjunct treatment for eating disorders: a qualitative enquiry of client perspectives
Source: BMC Complement Med Ther. 2024 Jun 24;24:245. doi: 10.1186/s12906-024-04514-1 (PMC11194889; doi:10.1186/s12906-024-04514-1)
Supplement: Supplementary file 1 — Supplementary Material 1. [file 12906_2024_4514_MOESM1_ESM.docx]

*Thematic Template for interviews regarding yoga as an adjunct for eating disorder treatment.*

- Overall research question: *What are the experiences and perspectives of yoga as an adjunct treatment to support eating disorder recovery?*
- Guiding questions for thematic analysis: *What are possible benefits of yoga for eating disorder recovery? What are enablers and barriers that impacted participation/engagement with yoga? What would I want from a yoga program?*

1. **Yoga’s capacity for healing and transformation**
   1. Physical
      1. Connecting with breath
      2. Gentle way to get endorphins
      3. Gentle movement
      4. Increase strength
      5. Increased flexibility
      6. Increased fitness
      7. Increased my bodies capability
      8. Slowing down my body
      9. Being present in my body
      10. Improved mood
      11. Reduced cravings
      12. Helped manage weight
   2. Psychological
      1. Connecting with emotions
      2. Mindfulness
      3. Helps me feel calm
      4. Not thinking about weight, food
      5. Relaxing
      6. Feeling safe in my body
      7. Occupies my mind positively
      8. Quietens my mind
      9. Improved acceptance
      10. Improved sense of autonomy
      11. Learnt to trust myself
      12. Increased self esteem
      13. Positive self-talk, positive affirmations
      14. Feel empowered
      15. Helped to develop sense of identity away from ED
   3. Spiritual
      1. Experience states of bliss
      2. Time to be grateful
      3. Time to reflect
      4. Cosmic experience
      5. Seeking something beyond the mundane
      6. Your spirit changes
   4. Social
      1. Connection to others
      2. Not the only one to experience ED
      3. Community
      4. Gives me a routine
      5. Can do it with my mum
      6. Can do it with friends
      7. Understanding that everyone is different
      8. Developed new friendships, relationships outside of ED
      9. Hobby/occupation that I participate in
      10. Seen by others as yogi (rather than just an ED)

**2. *My yoga journey - how I found yoga, the timing counts*?**

**(What happens and when – the intervention continuum)**

- - 1. Had done yoga before experienced ED
       1. Previous experience improved likelihood of giving yoga a go during treatment
          1. improved confidence in doing asanas
          2. understanding of potential benefits for MH
          3. understanding of mindfulness
          4. had insight into how may be beneficial for ED
          5. normalised activity within a fitness/health routine

**Early intervention**

- - 1. Introduced when in early stages of recovery
       1. Did it because only movement allowed
       2. Primarily introduced at inpatient setting
       3. Specific ED classes improved engagement
       4. Looking to connect with other people with ED (without having to talk)
       5. Health professional encouraged
          1. for safe movement
          2. for mindfulness

**Midway intervention**

- - 1. Introduced in middle stages of recovery
       1. Not sure if they’d like it
       2. Aware it may enhance recovery
       3. Looking to connect with other people with ED outside of therapy
    2. Who introduced participant to yoga and why
       1. Found it themselves
          1. as wanted to move

healthy movement (safe exercise)

unhealthy movement (to burn calories)

- - - 1. Did it because it was suggested by health professional
         1. Did it as good reintroduction back to exercise
      2. Friend encouraged
         1. Hobby to do together
         2. Normalised using yoga for stretching and relaxing
      3. Family encouraged
         1. Previous experience with yoga meant they saw potential MH benefits

**Longer term intervention**

- - 1. Engaged with yoga later in recovery
       1. Longer term presentations with chronic symptoms
       2. Engaged with yoga as last resort
       3. exhausted traditional MH services

***3a) What helps me engage in yoga***

Environment

- - 1. Access
       1. Studio close by helped
       2. Attended as inpatient
       3. Technology (youtube = increased access)
    2. Culture
       1. Relaxed environment
       2. Participants similar age
       3. Open and inviting
       4. Increased social connections
    3. Physical aspects
       1. Smell, noise, lighting all important
       2. No mirrors
  1. Teacher factors
     1. Training
        1. Trauma informed helps
        2. Language important
        3. An expert - substantial knowledge about yoga and eating disorders
        4. Openness around lived experience of mental health difficulties
        5. Understanding of stigma and its effects
     2. Relationship factors
        1. An existing relationship with that practitioner for trust
        2. Open and kind approach
        3. Feeling accepted and safe
        4. Offer additional support where appropriate
        5. Be given opportunities to keep coming back
     3. Familiar facilitation
        1. Get to know participants and build comfort
        2. Take things slowly, casually
        3. Helps manage paranoia
        4. Delicately approach more ‘intense’ experiences
        5. Meet and chat teacher before hand/after
        6. Similar approach each week that can be built upon
        7. Understanding it’s a practice (it is what it is)

**3b) What makes it difficult to engage in yoga**

- 1. Environment of yoga class

1. Issues with studio
   1. not welcoming
   2. mirrors
   3. equipment important to support safe and comfortable practice
   4. feeling out of place to other participants
      1. different clothing choice (tight clothing trigger)
      2. different ages
      3. different abilities
      4. different bodies shapes
   5. Access issues to yoga class
      1. Money

ii) Regional hard to find classes

- - 1. Lots of different types of yoga (confusing)
    2. Bikram – unhelpful for ED

iv) Family hesitant about non-medical approach

- 1. Psychological factors (impacting negatively on experience)
     1. Self-criticism
     2. Perfectionism (looking at others and comparing)
     3. Worried about judgement from others
     4. Increases my anxiety to be around others
     5. May increase negative body image
     6. Its spiritual / Its religious
     7. Previous negative experiences with yoga
        1. Its slow
        2. It doesn’t work
        3. It’s too hard
  2. Family/clinician concerns
     1. safety concerns
     2. Worry it might encourage calorie restriction
     3. Its not evidence based
     4. Its not traditional approach to health treatment

**4. What I would want from a yoga program? (Clinical Implications)**

1. Where
   - 1. Location to be close to home
     2. At ED service would increase convenience
     3. In community would help normalise
     4. Could use recording to help with practice outside of session
2. How
   - 1. Different formats
     2. Option of individual practice as well as group
     3. Guided and varied exercises, with flexibility
     4. Making it manageable and achievable (start slow)
     5. Resources to take home
     6. Keep it out of school hours
        1. Education before starting about yoga
           1. Understand impact of breath work
           2. Understand asana shapes
           3. Understand possible benefits
           4. One on one session before starting with group
     7. Smaller groups desired
        - 1. Openness and ‘Peer’ feeling, with bonding, as social support
          2. Talking and sharing afterwards
          3. Some people find group challenging
          4. Preparing people for the group
          5. Supported with a MH clinician and yoga teacher
          6. Similar ages
          7. Option to bring support person/family member
3. Who
   1. Teacher factors
      1. Training
         1. Trauma informed helps
         2. Language important
         3. An expert - substantial knowledge about yoga and eating disorders
         4. Openness around lived experience of mental health difficulties
         5. Understanding of stigma and its effects
      2. Relationship factors
         1. An existing relationship with that practitioner for trust
         2. Open and kind approach
         3. Feeling accepted and safe
         4. Offer additional support where appropriate
      3. Familiar facilitation
         1. Get to know participants and build comfort
         2. Take things slowly, casually
         3. Helps manage paranoia
         4. Delicately approach more ‘intense’ experiences
         5. Meet and chat teacher before hand/after
         6. Similar approach each week that can be built upon
   2. Helpful to have MH clinician in room too
      1. Can offer support before and after
      2. Can make link to MH treatment
      3. Can check in on how people are
      4. Provide supports afterwards
